# Life Years at Risk: A Population Health Measure from a Prevention Perspective

**DOI:** 10.3390/ijerph6092387

**Published:** 2009-09-03

**Authors:** Yuejen Zhao, Rosalyn Malyon

**Affiliations:** 1 Institute of Advanced Studies, Charles Darwin University, Darwin NT 0909, Australia; 2 Health Gains Planning Branch, Department of Health and Families, Northern Territory 0909, Australia; E-Mail:rosalyn.malyon@nt.gov.au

**Keywords:** health services needs and demand, risk assessment, health status indicators

## Abstract

This paper aims to present life years at risk (LYAR), a new measure of population health needs for primary, secondary and tertiary prevention, which classifies health outcomes by care type and distinguishes between positive and negative outcomes. It is determined by the probability of ill-health event, population size and life years lost, based on expected incidence, prevalence and mortality. The LYAR consists of two components: the observed LYAR, available using disability adjusted life years, and the avoided LYAR. Three examples are given to illustrate the calculation and application of the measure. The advantages, disadvantages and policy implications are also discussed.

## 1. Introduction

Population health refers to overall level and dispersion of health status in a defined population [1]. The assessment of population health needs is central to planning and providing health services to improve population health and utilize health resources effectively and efficiently [2]. Improving population health means both increasing overall level of health and reducing health gaps by eliminating or alleviating health hazards and preventing the occurrence of disease, injury, disability and premature death [3,4]. Accordingly, the purpose of health services can be considered to be loss aversion, and the extent of the risk of ill-health in the population indicates the level of needs for health care.

Healthy life years were proposed as a measure for population health in the 1960s [5], and further developments have included health indices [6–9], quality adjusted life years [10–12] and disability adjusted life years (DALYs) [13–15]. Of these measures, the DALY has become widely known as the standard unit of measure in burden of disease and injury studies (referred to hereafter as burden of disease studies). It can also be applied in the economic evaluation of health interventions [16]. The DALY aggregates the years of life lost (YLL) due to premature death and the years lost due to disability (YLD) into a single number. The YLD is calculated on the basis of the duration and severity weight of the disability (the relative preference for the disability state compared with full health and death) to enable compatibility with YLL [13].

Although there has been much criticism of the DALY [17–19], it represents a significant improvement in accuracy of health needs measurement, because DALYs combine both fatal and non-fatal outcomes and change the measurement from counting number of deaths to calculating years of healthy life lost [20]. DALYs have been widely used to quantify and compare population health needs as an indicator of where resources should be directed to, given the current level of access to health care. A recent burden of disease study for Indigenous Australians identified the Indigenous population as having a disproportionately large share of total DALYs [21]. While this suggests that they should be a priority for health care services, the full extent of their needs is unclear as the DALY only represents the negative health outcomes (mortality and morbidity) that arise despite past and continuing health investments. The DALY provides no information on the extent of positive health outcomes (avoided disease, injury, disability and death). The negative health outcomes are insufficient for resource allocation [22]. The inclusion of positive outcomes forms a legitimate part of health needs assessment, because continued investment may be needed to keep negative outcomes low. For example, renal dialysis can postpone death for patients with renal failure and reduce DALYs, but it does not mean less need for health care. Measuring total health needs would better inform decisions on resource allocation and identify whether gaps in health status between sub-groups in the population arise from a lack of investment, relative differences in investment or level of access to the services.

A further limitation of the DALY is its inability to distinguish between needs for different types of health care [17,23]. A disease may have a high share of total DALYs, but this does not inform on the relative impact of different care types. For example, acute hospital care may provide medical and surgical acute care to reduce mortality, but more efforts may be needed to prevent the occurrence of the disease and disability [24]. Such information could facilitate prioritization between different sectors of health care and assist in the comparison of different types of intervention.

This study seeks to explore the use of the DALY as a measure of relative health needs particularly where there are differences in levels of access to health care. The objective of this paper is to present a new measure: life years at risk (LYAR) that classifies DALYs in accordance with different health care types from a prevention perspective, and incorporates both positive and negative health outcomes. Examples of the calculation and application of the LYAR are provided, and its limitations and policy implications discussed.

## 2. Method

Concepts from preventive medicine were used to construct the LYAR measure. From this perspective, health services encompass three types of care: primary prevention, which reduces incidence of disease or injury; secondary prevention, which lessens and avoids disability; and tertiary prevention, which saves lives and delays deaths [25,26]. Table 1 summarizes the function, target population and intervention features for the three types of health care. As a measure of health needs for the three care types, the LYAR represents the expected number of healthy life years at risk of disease, injury, disability and premature death over a given period of time in a specified population (refer to last column of Table 1).

The LYAR is based on the notion of risk, which combines the probability of an event occurring and the consequence of its occurrence [27]. In terms of health, risk can be expressed as the probability of illness or injury occurring and the fatal or non-fatal health outcomes from that event [28,29]. Thus, the magnitude of the LYAR depends on the proportion of the population falling ill or being injured, the level and duration of disability or years of life prematurely lost as a result of those diseases and injuries. Thus, the LYAR can be determined quantitatively by

(1) LYAR = probability of the event × population × expected healthy life years lost due to the event

Like DALYs, the expected values for these variables can be ascertained from clinical and epidemiological evidence, disease and death registers, service activity data and expert opinion [13]. In regard to the type of health care, the LYAR consists of three components:

(2) Total LYAR= LYAR of incidence + LYAR of disability + LYAR of mortality

LYAR of incidence (LYAR(I)) for a disease or injury reflects health needs for primary prevention, which equals expected incidence of an uncomplicated condition multiplied by the duration of the condition times the severity weight. Expected incidence includes two elements: prevented and observed incidences. Thus,

(3) $$\begin{array}{l} \text{LYAR}(\text{I})\;=\;\text{expected}\;\text{incidence}\;\times \;\text{duration}\;\times \;\text{disability}\;\text{weight}\\ \;\;\;\;\;\;\;\;\;\;\;\;\;\;\;\;\;\;\;\;\;\;\;\;=\;(\text{prevented}\;\text{incidence}\;+\;\text{observed}\;\text{incidence})\;\times \;\text{duration}\;\times \;\text{disability}\;\text{weight}\\ \;\;\;\;\;\;\;\;\;\;\;\;\;\;\;\;\;\;\;\;\;\;\;\;=\;\text{observed}\;\text{incidence}\;\times \;\text{duration}\;\times \;\text{disability}\;\text{weight}\;/\;(1-\;\alpha (\text{I})) \end{array}$$

where attainment α (I) = prevented incidences/(prevented incidences + observed incidences) for 0 ≤ attainment ≤ 1, under the assumption that the prevented cases would have the same duration and disability weight as the actual cases observed. The observed part of LYAR(I) could be estimated using the YLD for uncomplicated cases from the burden of disease studies.

LYAR of disability (LYAR(D)) represents health needs for secondary prevention, which equals expected incidence of a disabling complication of the condition multiplied by the duration of the complication times the disability weight. For a steady-state population, prevalence is approximately equal to incidence times duration [30]. The expected disability prevalence comprises two elements: prevented and observed disability prevalence. Thus,

(4) $$\begin{array}{l} \text{LYAR}(\text{D})\;=\;\text{expected}\;\text{incidence}\;\text{of}\;\text{disabilities}\;\times \;\text{duration}\;\times \;\text{disability}\;\text{weight}\\ \;\;\;\;\;\;\;\;\;\;\;\;\;\;\;\;\;\;\;\;\;\;\;\;=\;\text{expected}\;\text{prevalence}\;\text{of}\;\text{disabilities}\;\times \;\text{disability}\;\text{weight}\\ \;\;\;\;\;\;\;\;\;\;\;\;\;\;\;\;\;\;\;\;\;\;\;\;=\;(\text{prevented}\;\text{disabilities}\;+\;\text{observed}\;\text{disabilities})\;\times \;\text{disability}\;\text{weight}\\ \;\;\;\;\;\;\;\;\;\;\;\;\;\;\;\;\;\;\;\;\;\;\;\;=\;\text{observed}\;\text{disabilities}\;\times \;\text{disability}\;\text{weight}\;/\;(1\;-\;\alpha (\text{D})) \end{array}$$

where attainment α (D) = prevented disabilities/(prevented disabilities + observed disabilities), assuming the prevented disabilities would have the same duration and disability weight as the actual disabilities observed. The observed LYAR(D) could be estimated using the YLD for complicated cases from the burden of disease studies.

LYAR of mortality (LYAR(M)) describes health needs for tertiary prevention, which is the expected YLL attributable to the condition and incorporates two elements: prevented and observed YLL. Thus,

(5) $$\begin{array}{l} \text{LYAR}(\text{M})\;=\;\text{expected}\;\text{YLL}\;=\;(\text{prevented}\;\text{YLL}\;+\;\text{observed}\;\text{YLL})\\ \;\;\;\;\;\;\;\;\;\;\;\;\;\;\;\;\;\;\;\;\;\;\;\;=\;\text{observed}\;\text{YLL}/(1-\alpha (\text{M})) \end{array}$$

where attainment α (M) = prevented YLL/(prevented YLL + observed YLL). The observed LYAR(M) could be estimated by the actual YLL from burden of disease studies.

While observed LYAR can draw largely on information from burden of disease studies, prevented LYAR is less directly observable. The following data sources may be helpful for estimating these components:

- Historical incidence, prevalence and mortality data;
- Epidemiological expectation, for example, dengue fever and influenza incidences in a neighboring state of similar climate or global epidemic pattern;
- Empirical estimation, for example, inferior health outcome in a neighboring population group without intervention;
- Service activity data;
- Risk factor analysis; and
- Early detection and screening data.

Burden of disease studies have been used to compare the health status of sub-groups within the population. If attainment (the proportion of prevented cases) is assumed to be equal across these sub-groups, each group’s share of total LYAR will be the same as its share of total DALYs. It can be shown that:

(6) $$\begin{array}{l} \text{LYAR}\;\text{Share}\;=\;\frac{\text{expected}\;\text{LYAR}}{\sum \text{expected}\;\text{LYAR}}\;=\;\frac{\left(\text{prevented}\;\text{LYAR}\;+\;\text{observed}\;\text{LYAR}\right)}{\sum \left(\text{prevented}\;\text{LYAR}\;+\;\text{observed}\;\text{LYAR}\right)}\\ =\;\frac{\text{observed}\;\text{LYAR}/(1-\alpha )}{\sum \text{observed}\;\text{LYAR}/(1-\alpha )}\;=\;\frac{\text{DALYs}}{\sum \text{DALYs}} \end{array}$$

where attainment α =prevented LYAR/(prevented LYAR + actual LYAR). If, however, attainment is less in some population groups, for example, there is less access to services or a greater degree of unmet needs, the DALY will be oversensitive to negative outcomes for these groups. This oversensitivity occurs because the actual LYAR prevented is smaller than that derived from uniform α, and the sub-group’s DALY share would be greater. Table 2 gives a hypothetical example to demonstrate the rationale why the DALY is oversensitive to negative health outcomes. The total true amount of ill-health is shown by the expected LYAR (column 1) and its share (column 2). If not all members of groups A and C have access to preventive care, but all of Group B does (column 3), then assuming equal effectiveness of the care, a smaller proportion of life years will be prevented in groups A and C (column 4). The observed number of actual life years, as captured in the DALY (column 5), would be greater for groups A and C and their DALY share (column 6) greater than it would have been (column 2), had access to preventive care being equal across the groups. In this sense, the DALY is more generous to disadvantaged groups with inadequate access to health care services.

Existing data for ischemic heart disease (IHD) and type 2 diabetes were used to illustrate the calculation and application of the LYAR. For IHD, incidence and mortality data were taken from Australian hospital morbidity and mortality databases [31]. The standard life expectancy and the disability weight for angina pectoris (0.105) were obtained from the 2003 Australian burden of disease study [32] with the expected duration by sex and age ranging from 2.4 to 24.5 years. For type 2 diabetes, the observed LYAR rates were derived from the Northern Territory (NT) and Australian burden of disease studies [32,33]. The disability weights and disease sequelae for type 2 diabetes were identical between the two studies. Due to multiple data sources in burden of disease studies, it was necessary to use DISMOD software to check and maintain the internal plausibility and mathematical consistency between incidence, prevalence, mortality and duration estimates [34]. The prevented component of LYAR was excluded as it only sought to demonstrate the split of LYAR by types of care for type 2 diabetes. The observed LYAR(I) was based upon uncomplicated cases of type 2 diabetes. The LYAR(D) was derived using the prevalence of retinopathy, cataract, glaucoma, diabetic foot, amputations, renal failure, neuropathy, peripheral vascular disease, IHD and stroke attributable to type 2 diabetes. The LYAR(M) was determined by underlying cause of death. The NT Indigenous population for the period 1999–2003 was derived on the basis of the total estimated resident population (ERP) and the experimental Indigenous ERP [35,36]. The national population was the Australian ERP in 2003 [37]. The rate ratios and their significances were inferred by using conventional statistical methods [38].

## 3. Results

During 2001–2002, the observed IHD incidences in Australia were estimated to be 48,700 and the prevented incidences were about 12,000 (based on historical incidence level prior to intervention) [31]. This data generated an observed IHD LYAR(I) during 2001–2002 of 33,600 and a prevented LYAR(I) of 3,800. Thus, total LYAR(I) would be 37,400 and the attainment factor for IHD primary prevention about 20%.

In 2006, the observed number of actual deaths attributable to IHD was about 23,000 with the prevented deaths approaching 73,000 [39]. This data generated an observed IHD LYAR(M) in 2006 of about 177,900 and a prevented LYAR(M) of 564,600. Thus, total IHD LYAR(M) in 2006 would be 742,500 and the attainment factor for IHD tertiary prevention about 76%.

The next example demonstrates the application of observed LYAR on type 2 diabetes. Table 3 shows the type 2 diabetes rate ratios of NT Indigenous LYAR per 1000 population compared to the Australian average by age groups and three types of care. The total LYAR per 1000 population for both NT Indigenous males and females were over four times the Australian rates. At all levels (age group, gender and type of care), the observed LYAR per 1000 population for the NT Indigenous population was greater than that for the Australian general population. The differences were of statistical significance (P < 0.01) except for LYAR(I) at ages 70 years and over. The rate ratios increased with descending age. The rate ratios for secondary prevention were greatest, over three times those for primary and tertiary prevention. The most striking differentials were in the age group 0–29 years for secondary and tertiary prevention.

## 4. Discussion

In line with the DALY, the LYAR measures the health needs of a defined population based on the probability of ill-health, the expected duration of illness, the presence of disability and reduced longevity [40]. When comparing sub-groups within the population, the DALY share for groups with relatively poorer access to health care is likely to be higher than might otherwise be the case using LYAR, which considers the whole need (positive and negative health outcomes). Such amplification would give greater emphasis to their relative circumstances and may be desirable depending upon the purpose of the evaluation. If policy makers are seeking evidence to spend additional resources, then the DALY share will direct resources towards the population with more negative outcomes, but it will offer no insight about what type of care is needed. If policymakers are evaluating the effectiveness of current spending, LYARs may be the better measure. By including both prevented and observed instances, the LYAR provides insight into the remaining burden of disease as well as the achievements from on-going investments by types of care. Effective investments in prevention would be expected to lead to a lower DALY because they will lower incidence, disability and/or mortality. This does not, however, mean a lesser need for health services if on-going services are required to maintain the current level of prevented cases. In contrast, the LYAR would not be affected because it shows the whole health need by including the prevented instances.

The division of the LYAR according to the three types of health care would allow assessment of the relative contributions of pre-emptive, curative and life-saving interventions [41]. This dimension of the measure may be valuable for comparing the benefits of interventions in different sectors. It would also show the effect that interventions may have on other care types. Effective primary prevention may also reduce the LYAR for secondary and tertiary prevention because it lowers the occurrence of the disease. Effective secondary prevention (i.e. treatment of disease and injury) can reduce the LYAR for tertiary care. Capturing these dynamics could better inform on the relative impact of interventions in different care sectors.

The prevention perspective of the LYAR can shift the focus beyond medical solutions and capture the impact on population health of other intercessions to improve education, employment, community infrastructure and other socio-economic factors, including poverty and the social and economic participation of people with disabilities. Furthermore, the LYAR can capture improvements in the functionality of disabled persons by reassessing the disability weight to reflect the change in the level and sequelae of disability associated with a disease or injury. Although rehabilitative services improve quality of life and prevent premature death, they do not, however, prevent the condition.

Health funding in Australia has largely overlooked mortality and morbidity [42]. This omission may be due to concerns that the mortality and DALY measures do not recognize the full needs for health care. The LYAR is a step toward an alternative measure that can more inform funding decisions by presenting a full picture of health needs.

There are some limitations to the LYAR measure. First, it would require information on both observed and prevented disease and injury. Thus, its data needs are considerable. While data on observed cases may be available, estimating prevented cases may be more difficult as it may not be possible to readily observe their numbers. Historical incidence may be also difficult to estimate accurately if appropriate and reliable records have not been kept. Second, LYAR will be vulnerable to errors in assumptions and interpretations of the occurrence and experience of disease and injury. For example, rehabilitative services may not change the LYARs if the disability weights only reflect diagnoses, not functionality and quality of life. Third, the LYAR applies the concept of disability weights from the DALY so may be subject to similar criticisms to the DALY [18]. Further research is needed to consider the prospect of wider application and refinement of the population health needs measure that covers both positive and negative health outcomes.

## Figures and Tables

**Table 1.** Health needs measurement from a prevention perspective.

**Health care type**	**Health care aim**	**Target population**	**Intervention features**	**Proposed health needs measure**
Primary prevention	Reduce incidence	Population at risk	Eliminate or alleviate causes, risk factors or determinants of the condition; disease surveillance; immunization, education, health legislation	LYAR of incidence of disease or injury
Secondary prevention	Reduce disability	Patients with early stage of the condition	Cure disease or mitigate impairments by early detection, prompt diagnosis and treatment	LYAR of disability
Tertiary prevention	Reduce mortality	Patients with late stage of the condition	Maintain function or prolong survival; life-saving interventions, surgeries and procedures; rehabilitative and palliative care	LYAR of mortality

Note: LYAR=life years at risk. Modified from Reference [25].

**Table 2.** An hypothetical example (in life years, assuming effectiveness α = 50%).

	**Expected LYAR (‘000)**	**LYAR share**	**Treated LYAR (‘000)**	**Prevented LYAR (‘000)**	**Actual DALYs (‘000)**	**DALY share**
	(1)	(2)	(3)	(4) = (3) × α	(5) = (1) – (4)	(6)
**Group A**	30	5%	10	5	25	8%
**Group B**	500	83%	500	250	250	76%
**Group C**	70	12%	36	18	52	16%

**Table 3.** Observed life years at risk of type 2 diabetes per 1,000 population by age group and type of care, Northern Territory Indigenous population 1999–2003 vs Australia 2003.

	**NT Indigenous**		**Australia**		**Rate ratio**	

	**Male**	**Female**	**Male**	**Female**	**Male**	**Female**

**LYAR (I) for primary prevention**						
0–29	4.9	8.0	0.2	0.5	28.9^*^	17.6^*^
30–49	17.4	21.5	6.4	5.1	2.7^*^	4.2^*^
50–69	18.5	20.5	9.5	7.9	2.0^*^	2.6^*^
70+	8.1	10.2	6.6	8.0	1.2	1.3

Total	9.0	12.6	4.4	4.1	2.0^*^	3.1^*^

**LYAR (D) for secondary prevention**						
0–29	2.7	2.4	0.0	0.0	1279.2^*^	163.8^*^
30–49	44.8	36.1	0.6	0.7	73.9^*^	51.9^*^
50–69	113.6	93.2	4.2	3.2	27.2^*^	29.1^*^
70+	49.7	42.7	9.7	9.7	5.1^*^	4.4^*^

Total	22.2	20.1	1.8	1.9	12.3^*^	10.7^*^

**LYAR (M) for tertiary prevention**						
0–29	0.2	0.4	0.0	0.0	134.9^*^	113.6^*^
30–49	18.4	15.8	0.5	0.3	38.6^*^	58.6^*^
50–69	76.3	69.8	6.9	3.0	11.0^*^	23.6^*^
70+	122.5	117.5	36.6	33.7	3.4^*^	3.5^*^

Total	12.4	13.0	4.5	4.2	2.8^*^	3.1^*^

**LYAR for total health care**						
0–29	7.8	10.8	0.2	0.5	45.4^*^	22.6^*^
30–49	80.6	73.3	7.5	6.1	10.8^*^	12.0^*^
50–69	208.4	183.5	20.6	14.0	10.1^*^	13.1^*^
70+	180.3	170.3	52.9	51.4	3.4^*^	3.3^*^

Total	43.6	45.7	10.7	10.2	4.1^*^	4.5^*^

**Estimated resident population**						
0–29	94585	89180	4128384	3966432		
30–49	33903	35809	2933508	2974180		
50–69	11174	12808	2030435	2022961		
70+	2109	2893	779315	1046254		

Total	141771	140690	9871642	10009827		

Note: LYAR = life years at risk, I = Incidence, D = Disability, M = Mortality; NT = Northern Territory, Australia;

* P < 0.01.
