# Editor’s Note – Received Date Error, *Int. J. Environ. Res. Public Health* 2009, *6*, 2387–2396

**DOI:** 10.3390/ijerph6112762

**Published:** 2009-11-03

**Authors:** Gardenia You

**Affiliations:** MDPI Beijing Office, Floor 4, Chelifen Village Committee Office Building, Liyuan Town, Tongzhou District, 101101 Beijing, China; E-Mail: gardenia.you@em.mdpi.org; Tel.: +86 10 605 288 33; Fax: +86 10 605 247 74

*Editor’s Note* added on 3 November 2009: The received date was wrong in *Int. J. Environ. Res. Public Health* **2009**, *6*, 2387–2396. It should be 29 July 2009 instead of 29 July 2008.
